# Evoking artificial speech perception through invasive brain stimulation for brain-computer interfaces: current challenges and future perspectives

**DOI:** 10.3389/fnins.2024.1428256

**Published:** 2024-06-26

**Authors:** Yirye Hong, Seokyun Ryun, Chun Kee Chung

**Affiliations:** ^1^Department of Brain and Cognitive Sciences, College of Natural Sciences, Seoul National University, Seoul, Republic of Korea; ^2^Neuroscience Research Institute, Seoul National University Medical Research Center, Seoul, Republic of Korea

**Keywords:** brain-computer interfaces, electrocorticography (ECoG), speech processing, artificial perception, invasive brain stimulation, direct cortical stimulation

## Abstract

Encoding artificial perceptions through brain stimulation, especially that of higher cognitive functions such as speech perception, is one of the most formidable challenges in brain-computer interfaces (BCI). Brain stimulation has been used for functional mapping in clinical practices for the last 70 years to treat various disorders affecting the nervous system, including epilepsy, Parkinson’s disease, essential tremors, and dystonia. Recently, direct electrical stimulation has been used to evoke various forms of perception in humans, ranging from sensorimotor, auditory, and visual to speech cognition. Successfully evoking and fine-tuning artificial perceptions could revolutionize communication for individuals with speech disorders and significantly enhance the capabilities of brain-computer interface technologies. However, despite the extensive literature on encoding various perceptions and the rising popularity of speech BCIs, inducing artificial speech perception is still largely unexplored, and its potential has yet to be determined. In this paper, we examine the various stimulation techniques used to evoke complex percepts and the target brain areas for the input of speech-like information. Finally, we discuss strategies to address the challenges of speech encoding and discuss the prospects of these approaches.

## 1 Introduction

The application of invasive brain stimulation has been widely employed in clinical settings to investigate brain function and modulate neural activity. In this review, we utilize the term “*modulation*” to denote alterations in neural activity that produce changes in behavior, such as the reduction of involuntary movements in patients with motor dysfunction or the enhancement of memory function. Conversely, “*encoding*” pertains to the generation or introduction of novel perceptions into the brain through electrical stimulation, essentially creating artificial perceptions rather than modifying existing functions.

Since the mid-19th century, brain stimulation has been performed to evoke various perceptions. In 1870, Fritsch induced somatotopic motor movements using the invasive electrical stimulation of the canine cortex (Fritsch, 1870). In the 20th century, stimulation was used to induce new perceptions or modify existing cognitive functions in mammals (Asanuma and Rosen, 1972; Andersen et al., 1975). Penfield, one of the pioneers of brain stimulation, evoked various hallucinations, such as flashbacks and forced conceptual thoughts, by stimulating multiple regions of the human cortex (Penfield and Perot, 1963). Specifically, the motor and somatosensory cortices have been thoroughly investigated using brain stimulation, and the organization of neuronal responses in these areas has been extensively mapped, also known as somatotopy. The somatotopic map, or the somatosensory “homunculus” was introduced by Penfield, who summarized the cortical and subcortical stimulation data from 126 operations to map out each area of the human motor and somatosensory cortices (Penfield and Boldrey, 1937). The visual cortex has also been stimulated to create visual perception, with the intention of developing visual cortical devices for blind subjects or those with visual impairments (Liu et al., 2022). In the 90’s, Bak introduced the potential for producing visual perceptions using intracortical microsimulation (ICMS) of the human occipital cortex (Bak et al., 1990). The stimulation of the motor-sensory cortex or visual cortex has been extensively investigated since the mapping and localization of these regions to their specific functions can be easily attained. However, higher cognitive functions such as memory and language have remained difficult to encode in the human brain. Thus, novel approaches to stimulation need to be applied rather than conventional stimulation methods that have been widely adopted to map and study speech-related regions. In this paper, we discuss the current difficulties and constraints, the current approaches to evoking intricate cognitive perceptions with brain stimulation, and the critical brain regions involved in speech perception. Finally, this study delves into some recent technology that can be used to circumvent the current challenges encountered in speech encoding and explores the prospects of these approaches.

## 2 Background

Electrical stimulation has traditionally been utilized for the functional mapping of the cortex prior to resective brain surgery. This method involves establishing a correlation between the location of the cortex and its specific function. Epilepsy patients undergoing awake surgery have provided a unique opportunity to investigate the cortical function and structure of each brain region. Neurosurgeons Penfield and Ojemann were the pioneers in mapping the eloquent cortices to investigate the neurophysiological correlates of language, memory, and other higher cognitive functions (Penfield and Rasmussen, 1950; Penfield and Perot, 1963; Ojemann and Dodrill, 1985; Ojemann et al., 1988; Ojemann, 1990). Since then, various regions of the cortex have been mapped and studied, including the auditory, memory, and language regions, all of which have been investigated through functional mapping.

The field of artificial somatosensation has garnered significant attention in recent years due to its potential applications in treating patients with various sensorimotor impairments such as spinal cord injury or stroke. Brain-computer interfaces (BCIs) and neural prostheses have been identified as effective tools for delivering artificial somatosensory input to these patients. The somatosensory function has been extensively studied in both animals and humans, with researchers discovering that somatosensation can be evoked by stimulating the cortical surface, and their function can be mapped by connecting brain regions to corresponding tactile sensations in different body parts, also known as somatotopy (Penfield and Boldrey, 1937; Walter et al., 1992; Rech et al., 2016; Isitan et al., 2020; Ryun et al., 2023). In an awake-craniotomy, Cushing performed electrical stimulation of the post-central gyrus and evoked somatosensory percepts such as “numbness” and “twitching” as well as generating detailed maps of the somatosensory cortex, including the sensory-motor homunculi (Cushing, 1909). Non-human primates have been used to evoke artificial sensations as well. Intracortical microelectrodes were used in non-human primates to discriminate between different stimulation parameters in the somatosensory cortex (Romo et al., 1998; O’Doherty et al., 2011). In contrast to the electrodes placed on the surface of the brain, ICMS has been found to produce more intricate somatosensation rather than a vague “numbness” or “tingling” that was induced in the past (Penfield and Boldrey, 1937; Penfield and Rasmussen, 1950; Roux et al., 2018). In a texture discrimination task, monkeys were able to discriminate different artificial tactile stimulation induced by ICMS in the S1 cortex (Romo et al., 1998). This task showed that artificial somatosensation can be fine-tuned and controlled through electrical stimulation. In more recent studies, surface electrodes have been used to evoke various somatosensations in the upper limb (Flesher et al., 2016; Hiremath et al., 2017). Although the somatosensory regions have been mapped and localized extensively through stimulation, artificially evoked somatosensory perception and its relation to natural somatosensation by sensory inputs have yet to be discovered (Kirin et al., 2019).

Electrical stimulation of the visual cortex induces “phosphenes,” which are perceptions of small spots of light (Dobelle, 2000; Winawer and Parvizi, 2016; Bosking et al., 2017). In the 1900s, Penfield and Rasmussen evoked visual perceptions of phosphenes when stimulating the occipital cortex (Penfield and Rasmussen, 1950). DCS near the occipital cortex, specifically occipitotemporal and occipito-parietal areas, evoked visual perceptions such as patterns, spots, shapes, flashes of light, colors, and phosphenes in the external world (Mégevand et al., 2014; Andelman-Gur et al., 2020). This is not to be confused with complex speech and déjà vu related perceptions, where stimulation evokes scenes or people inside the mind. Visual perceptions involve external visual imagery, where the eyes see various images in the external world. Recently, visual cortical prostheses (VCP) have been developed, which are devices that restore partial visual function to patients who have retinal damage. Some VCPs are in clinical trials, and many prototypes are under development for clinical use (Troyk et al., 2003; Tehovnik and Slocum, 2013; Niketeghad et al., 2019). However, similar to somatosensory perception, the problem of reliably and repeatedly generating the same visual perceptions in every subject has not been solved (Bosking et al., 2022).

Auditory perception has been evoked by stimulation of the primary auditory cortex, including Heschl’s gyrus, and various areas of the temporal lobe, particularly the superior temporal gyrus. Previous studies have shown that stimulation of the superior temporal gyrus evokes auditory hallucinations (water dripping, buzzing, human voices) (Selimbeyoglu and Parvizi, 2010; Leonard et al., 2019). Furthermore, the deep brain stimulation of Heschl’s gyrus can evoke the perception of distal tones (Donovan et al., 2015). Recently, studies have shown that electrical stimulation of the planum temporale improves speech perception in noise, which shows applications of brain stimulation in restoring hearing. However, despite the vast research in the stimulation of auditory regions, the possibility of creating speech–like perceptions is still yet to be determined. Creating elaborate speech sounds through brain stimulation has been proven difficult since simple auditory perceptions cannot be classified as speech. Fine-tuning the evoked responses of stimulation and reliably generating consistent auditory hallucinations may be one of the key tasks to solve when eliciting speech-like perceptions in the human brain. Much further study needs to be done to encode intricate speech sounds in the human brain and ultimately achieve similarity to actual human speech.

Hallucinations are vivid perceptions created in one’s mind that can be evoked during brain stimulation in the absence of other external stimuli. Penfield distinguished illusions and hallucinations, which are both categorized as experiential phenomena or vivid subjective experiences evoke by cortical stimulation that is usually related to one’s past (Mullan and Penfield, 1959; Penfield and Perot, 1963; Gloor, 1990; Sjöberg, 2023). Illusions are distortions of real perceptions or sensory stimuli, such as echoes or distortions of external objects, whereas hallucinations can be defined as vivid percepts that are experienced in the environment, such as hearing noises or seeing faces (Braun et al., 2003; Elliott et al., 2009; Jaroszynski et al., 2022). Visual hallucinations have been electrically evoked in various parts of the brain using invasive stimulation. These can be mental visual imagery, which are subjective perceptions of thoughts and images in the mind, or hallucinations that are seen in the external world. These hallucinations are usually related to one’s past experiences. One of the first reports of visual hallucinations evoked through brain stimulation is from Forster (Forster, 1936). Six patients reported hallucinations of figures such as animals and persons with stimulation of the superior lateral occipital lobe. Visual hallucinations could be evoked in other brain regions, such as the frontal lobe. In 2000, Blanke elicited vivid visual hallucinations during stimulation of the left frontal lobe in two epilepsy patients undergoing resection surgery (Blanke et al., 2000). This study shows that intricate visual perceptions could be evoked in various brain regions, further indicating that complex perceptions involve a network of areas rather than being localized in one region. The stimulation of various cortical regions resulted in the input of various new sensory percepts in the human brain. However, the input of speech-like information with electrical stimulation still needs to be explored since speech signals require the activation and integration of multiple sensory and perceptual level processes, from the input of acoustic information to memory recall (Pisoni, 1993; Lim et al., 2019).

## 3 Challenges of inducing artificial speech perception with direct cortical stimulation

Despite the ongoing studies of neuroscience, the neurophysiological mechanism behind stimulation is still poorly understood (Kucewicz et al., 2022). The cellular, molecular, and clinical effects of stimulation are being unraveled (Jakobs et al., 2019). Unlike other cognitive functions like motor or visual perceptions, speech processing activates a vast network of brain regions, spanning sensorimotor, limbic, and executive networks (Kucewicz et al., 2022). Thus, the exact function and relationship between networks that contribute to speech perception is still largely unknown. The dual-stream model of speech processing has been widely supported in recent years, where the ventral and dorsal streams of speech processing act independently of one another (Hickok and Poeppel, 2007; Hamilton et al., 2021). Speech perception comprises a number of cognitive functions but fundamentally involves the recognition and processing of speech signals and subsequent comprehension of semantic, grammatical, and thematic structures in speech (Friederici, 2011). Language processing engages complex physiological mechanisms spanning various levels of brain organization, from individual cells to local assemblies and large-scale distributed networks across multiple cortical and subcortical regions (Kucewicz et al., 2022). Intracranial recordings and DCS of the human brain offer a unique and powerful means to investigate the role of specific brain regions involved in speech and other cognitive functions; intracranial recordings offer high spatiotemporal resolution and high signal-to-noise ratio due to their direct contact with the cortex, which is otherwise unavailable in its non-invasive counterparts such as fMRI and electroencephalography (EEG) (Mukamel and Fried, 2012). A recent investigation using invasive brain stimulation attempted to uncover the function of these networks by visualizing the white matter that composes the inferior fronto-occipital fasciculus (IFOF), which is a white matter tract within the orbito-frontal region associated with semantic language processing. Stimulating the electrodes implanted near this network elicited complex visual hallucinations in two patients, which suggests that stimulation location and the pathways related to language processing play a big role in the mapping of cognitive functions (Andelman-Gur et al., 2020). Despite these ongoing stimulation studies uncovering the mechanisms and regions involved in speech, evoking artificial speech-like perceptions with DCS poses several key challenges: the complexity of brain areas involved in speech cognition, the inhibition or decrease in neural activity during electrical stimulation on language areas, and the lack of knowledge regarding the mechanisms of stimulation.

Mapping and correlating the retrieval, storage, and formation of speech to areas of the brain has been one of the most challenging tasks. It is widely known that stimulation of the visual cortex evokes various visual perceptions, while stimulation of the parietal lobe evokes somatosensory perceptions (Silverstein, 2012; Bosking et al., 2022). These visual, somatosensory, and motor regions of the human brain have been extensively mapped since Penfield, where stimulation of these areas usually leads to positive perceptions related to that specific region. However, stimulation of the temporal lobe or areas that are related to speech processing does not always result in the creation of positive phenomena. More often than not, stimulation of language or auditory cortices resulted in the inhibition of speech rather than eliciting a positive language phenomenon (Selimbeyoglu and Parvizi, 2010). Electrical stimulation mapping of the language areas of the brain has mostly resulted in the inhibition or decrease in neural activity; in most cases, stimulation of the eloquent cortex has led to patient reports of anemia, paraphasia, aphasia, and other kinds of speech errors or inhibitions in speech abilities (Penfield and Rasmussen, 1950; Penfield and Perot, 1963; Ojemann and Dodrill, 1985; Ojemann et al., 1988; Ojemann, 1990; McIntyre et al., 2004; Selimbeyoglu and Parvizi, 2010; Lu et al., 2021).

It is often assumed that electrical stimulation of a specific cortical area will consistently elicit the same neurophysiological and behavioral responses upon stimulation. In practice, however, electrical stimulation of certain patients elicits complex perceptions, while stimulation of the same areas in a different patient leads to inhibition of speech or no effect at all. Functional mapping of language areas shows inter-subject variability where each patient shows different responses when stimulation of the same cortical area with the same stimulation parameters (Duffau et al., 2002; Corina et al., 2010; Schumacher et al., 2019; Jaroszynski et al., 2022). In studies that used identical parameters (frequency, amplitude, stimulation duration, etc.) on multiple patients, stimulation of the same cortical or subcortical region in one patient did not elicit the same response in a different patient. This variability across subjects is what makes evoking consistent perceptions through invasive stimulation difficult; every patient’s neural structure is unique, and these cognitive networks are located in slightly different areas. Direct cortical stimulation (DCS) results in a complex response from the underlying neural networks, leading to heterogeneity in neural, cognitive, and behavioral effects even when using the same stimulation parameters (Borchers et al., 2011). Even stimulation of identical sites on the same patient at different times may lead to different perceptions (Roux et al., 2017). Known as Intra-subject variability, stimulation of identical regions on the same patient at different times evokes different perceptions, making the encoding of perception through stimulation even more challenging. This inter- and intra-subject variability can be attributed to various factors. The stimulation causes the corresponding brain region to undergo significant fluctuations in excitability, which are reflected by the phase of ongoing low-frequency oscillations, particularly in the theta frequency range (Lakatos et al., 2005; Moheimanian et al., 2021). Another reason may be due to a concept called “mixed selectivity,” where neurons exhibit responses that are influenced by a wide range of non-linear combinations of task-relevant variables (Fusi et al., 2016). Finally, stimulation of white matter tracts elicits a different behavior than stimulation of the surrounding gray matter. Studies show that white matter proximity to the stimulation point has a substantial impact on the behavioral and physiological responses of each patient (Duffau, 2015; Paulk et al., 2021, 2022). In fact, a recent study shows that stimulation at the boundary between gray and white matter elicited a larger response locally (<15 mm to stimulation site), whereas white matter stimulation evoked a larger response than gray matter stimulation at distant sites (>15 mm to stimulation site) (Paulk et al., 2022).

The vast interconnected network involved in higher-order perceptions such as memory and speech proposes another challenge when evoking perceptions through invasive stimulation techniques. Stimulation studies suggest that speech and higher-order cognitive perceptions involve the activation of widely distributed neural networks (Sanai et al., 2008; Elliott et al., 2009; Selimbeyoglu and Parvizi, 2010; Andelman-Gur et al., 2019). Various perceptions of mnemonic, affective, and speech content were evoked in the occipital and temporal lobes and also the frontal lobe during invasive stimulation (Gloor, 1990; Elliott et al., 2009; Selimbeyoglu and Parvizi, 2010; Andelman-Gur et al., 2019). A recent simulation study using depth electrodes in the frontal, temporal, parietal, and occipital areas shows that complex perceptions are evoked during stimulation of the inferior frontal-occipital fasciculus (IFOF) (Duffau et al., 2002; Andelman-Gur et al., 2019). Furthermore, a study using intracranial neural recordings and stimulation across the entire human auditory cortex indicates that speech perception and language processing are not in a hierarchical organization where one area is activated then is followed up with another region of activation, but rather is activated simultaneously during language tasks (Hamilton et al., 2021). Specifically, the superior temporal gyrus processes speech information independent of primary auditory regions (Hamilton et al., 2021). Recently, a DCS study showed the complex disruption and activation of neural networks during stimulation; this sheds light on the complex functional organization of the human connectome and how behavioral reports differ even when stimulating the same region in different patients (Duffau, 2020). A meta-analysis study of fMRI and other functional neuroimaging literature shows that natural speech perception relies on dynamic neural networks and that it cannot be defined in a distinct area like visual or motor perception (Vigneau et al., 2006). Thus, language and other cognitive functions are distributed in large brain areas and involve the complex networks of the human connectome; simply stimulating one brain region may not produce the desired effect of eliciting speech perception.

## 4 Target brain regions for speech perception

Speech perception relies on the transformation of acoustic information into linguistic representations. It is a multimodal process involving not only the primary auditory cortex and the temporal lobe but also other areas of the brain, such as the visual, motor, somatosensory, and prefrontal cortices. Therefore, participation in multiple areas is essential for evoking elaborate percepts in the human brain. Brain regions involved in responding to auditory stimuli are also involved in speech perception (Démonet et al., 2005; Hamilton et al., 2021). The human auditory cortex is a vast area in the temporal lobe, including the superior temporal gyrus, planum temporale, and Heschl’s gyrus. Stimulation of these areas is known to evoke auditory hallucinations and other kinds of complex percepts (Jaroszynski et al., 2022).

Although the visual cortex is not directly associated with language processing, visual inputs influence speech perception. Visual inputs bias speech perception either positively or negatively, and dynamic temporal visual stimuli can improve speech perception in noise (Sumby and Pollack, 1954; Yuan et al., 2021). The human brain has neural pathways dedicated to visual speech perception, allowing for the perception of speech through visual cues (Bernstein and Liebenthal, 2014). Recent studies show that visual speech can enhance auditory speech recognition and processing, indicating the presence of cross-modal interactions between the visual and auditory cortex (Arnal et al., 2009; Karas et al., 2019). Furthermore, cortical stimulation of the temporal-parietal-occipital junction (Brodmann area 19) created complex visual hallucinations of a cat in boots while the patient was reading a text without pictures from *Puss in Boots* by Charles Perrault (Schulz et al., 2007). This indicates that the activation of speech areas during reading tasks modulates the creation of complex visual hallucinations during stimulation. Moreover, studies have demonstrated that visual speech can enhance auditory speech recognition and processing, indicating the presence of cross-modal interactions between the visual and auditory cortex (van Wassenhove et al., 2005). These multisensory interactions play a crucial role in modulating activity throughout the speech perception network, showcasing the integration of visual and auditory information at various levels of language processing.

The motor cortex is widely known for its role in speech articulation and production. However, recent studies show that motor circuits are also involved in perception of speech sounds and subsequent language comprehension (D’Ausilio et al., 2009). For instance, the superior and inferior regions of the ventral motor area are activated during speech-listening tasks (Cheung et al., 2016; Lima et al., 2016). An fMRI study showed that frontal motor areas are activated during speech perception and production. Perception of speech sounds was associated with activity in the superior ventral premotor cortex, while articulation was associated with the primary motor cortex but not perception (Wilson et al., 2004). This pattern was confirmed by other neuroimaging studies; the complexity of speech perception showed a positive correlation with activity in the left ventral premotor cortex; and as complexity of the perceived speech increased, so did the activity in that cortical region (Tremblay and Small, 2011). In intracranial EEG studies, the ventral premotor cortex was active during natural speech perception tasks but not in the primary motor cortex (Cogan et al., 2014; Cheung et al., 2016; Glanz et al., 2018).

Recently, there has been more and more evidence that the inferior parietal lobule and somatosensory cortex also contribute to speech perception, known as the “somatosensory theory of speech perception” (Franken et al., 2022). Geschwind, a behavioral neurologist, first suggested the role of the angular gyrus in silent reading (Anderson et al., 1999). This Wernicke-Geschwind model, which suggests that the arcuate fasciculus is the main connection between Broca’s and Wernicke’s areas, is currently obsolete, but recent studies suggest the parietal lobule’s influence in speech processing. Lesion studies show that damage to the parietal lobe leads to impairment in speech tasks (Caplan et al., 1995; Kim et al., 2019; Rogalsky et al., 2022). A recent large-scale lesion study revealed that injury to the left supramarginal gyrus was associated with impairment in an auditory nonword discrimination task (Rogalsky et al., 2022). In another study, failures in phoneme discrimination and identification occurred in patients with injury to the left supramarginal gyrus and parietal operculum. These lesion studies suggest that the parietal lobe plays a role in auditory speech perception. Furthermore, the input of auditory stimuli also increases activity in these regions. Frequency-dependent activity in the parietal operculum was elicited during an auditory frequency discrimination task (Pérez-Bellido et al., 2018). Moreover, speech features can even be decoded from activity in the post-central gyrus during auditory listening tasks with fMRI recordings (Correia et al., 2015). Activity in the inferior part of the somatosensory cortex increased in response to auditory stimuli and responded differently to the place and manner of articulation of the auditory stimulation (Correia et al., 2015). This suggests that the somatosensory cortex encodes features of speech signals and passive speech perception. A fMRI study also showed activity in somatosensory regions during listening tasks, specifically in the pre- and post-central gyri (Arsenault and Buchsbaum, 2016). Furthermore, intracranial EEG recordings during a discrimination task of mandarin tones with English-speaking participants revealed high gamma activity in motor, premotor, and somatosensory areas, as well as the superior temporal gyrus (Yi et al., 2021).

**FIGURE 1:**
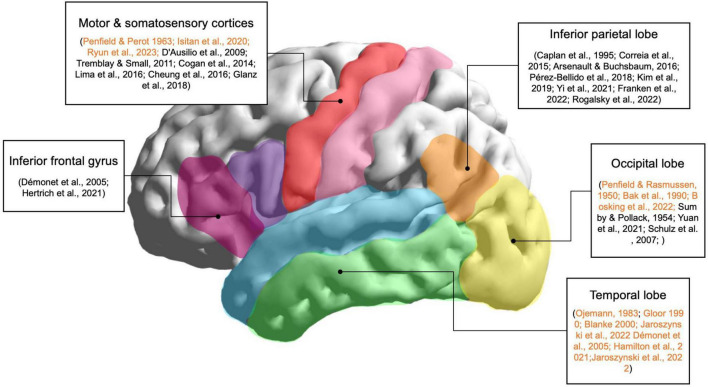
Main brain regions involved in speech perception. Lateral view of the left hemisphere of the human brain and the corresponding areas related to speech perception according to neuroimaging, EcoG, intracranial electroencephalography (iEEG), and stimulation studies. These regions are target areas for evoking speech-like perceptions using invasive brain stimulation techniques. Stimulation studies are highlighted in orange.

Thus, the visual, parietal, and motor cortices are all areas to consider for the induction of speech-like perceptions with direct cortical stimulation, as shown in Figure 1. Since these areas are involved in natural speech perception, they are promising target areas for stimulation; the simultaneous neural excitation of these areas may lead to speech-like complex perceptions that may not be evoked solely by stimulation of the temporal lobe. Although these areas may not directly lead to speech-like phenomena when stimulated, multi-parameter and multi-site stimulation of these areas may lead to promising results.

## 5 Various approaches to the encoding of speech-like perceptions

There are various possible methods for generating artificial speech-like percepts in the human brain. To induce diverse sensory and higher cognitive perceptions, fine-tuning of current frequency, amplitude, stimulation duration, pulse duration, and pulse shape is needed. To determine the most effective stimulation protocols, the underlying biological mechanisms governing cortical stimulations require in-depth investigation (Caldwell et al., 2019). In an intracranial EEG study examining the effects of different stimulation parameters on somatosensory and motor cortices, an increase in amplitude and frequency led to a heightened intensity of perceived somatosensation (Hiremath et al., 2017). Additionally, the nature of sensation experienced by patients varied in relation to the employed pulse width. These findings suggest that modulation of stimulation parameters can encode various information that would not be possible using conventional stimulation paradigms. Conventional paradigms use a set amplitude, frequency, and pulse width and do not examine the effects various parameters have on the patient’s subjective perceptions. A recent large-scale study involving 106 epilepsy patients revealed that diverse stimulation parameters yielded distinct neural activity patterns (Mohan et al., 2020). This study claims that high-frequency stimulation resulted in excitatory phenomena more often compared to conventional low amplitude, low-frequency stimulation. Whereas conventional approaches to stimulation and functional mapping of language regions have mostly resulted in the inhibition or decrease in neural activity, this research indicates that specific parameters foster enhanced neural excitation in regions implicated in higher cognitive functions. Further comprehensive stimulation studies utilizing a spectrum of stimulation parameters are imperative in designing protocols for eliciting speech-like perceptions.

Multi-site stimulation (MSS), which involves the simultaneous stimulation of multiple brain regions, is an emerging technique that has shown promise in improving the efficacy of invasive brain stimulation and speech encoding. Recent findings reveal targeting multiple nodes of brain networks simultaneously may play a key role in neuromodulation and the encoding of various information in the brain (Gonen et al., 2017; Hong et al., 2023). MSS was first used in cortical mapping during awake surgery for brain tumor resections or in drug-resistant epilepsy patients (Gonen et al., 2017). A recent study showed that identifying the nodes that show activity during working memory tasks and stimulating two nodes simultaneously results in increased performance compared to single-site stimulation (Alagapan et al., 2019). In 2022, stimulation of the visual cortex allowed the encoding of multiple phosphenes, whereas, in single-site stimulation of the visual cortex, only single phosphenes could be evoked (Bosking et al., 2022). However, the MSS of the temporal lobes to evoke complex perceptions has not been fully studied. MSS allows for the input of current in various regions of the brain simultaneously, mimicking the dynamic neural activity that occurs during speech. Since the exact neural correlates of electrical cortical stimulation are still under debate, MSS shows promise in uncovering the mechanisms behind cortical stimulation and allowing for the input of diverse information in the brain.

The response evoked by stimulating the same brain region exhibits significant variability among different subjects, a phenomenon referred to as inter-subject variability (Borchers et al., 2011). Thus, brain models need to be developed in order to create patient-specific stimulation guidelines and standardization of stimulation parameters. Recent studies report various brain models that accurately predict the effects of stimulation on an individual level (Yang et al., 2021). This study developed an input-output model that predicts the dynamic nature of brain networks and how they respond to stimulation. Another study predicts stimulation effects using high-resolution virtual brain models that mimic the spatiotemporal responses in actual neural fiber pathways (An et al., 2022). Personalized modeling of stimulation effects may be able to overcome the challenges of inter- and intra-subject variability in stimulation responses by predicting the outcomes before stimulation.

Closed-loop stimulation, where neural data is decoded in real-time from the human brain, and then the subsequent data is used for stimulation, is another method to optimize speech encoding. One of the first successful closed-loop stimulation systems was developed using a non-human primate model; this system utilizes ICMS to provide somatosensory feedback. In this study, closed-loop stimulation improved the decoding accuracy and response time of the BCI system compared to open-loop stimulation, suggesting that closed-loop stimulation may offer more precise and effective BCI control (Klaes et al., 2014). Recently, closed-loop stimulation was used to improve memory encoding as well. The study recorded and analyzed neural activity using electrocorticography (ECoG) electrodes, creating patient-specific models of neural activity based on memory performance and using those models to target stimulation to the lateral temporal cortex in real-time (Ezzyat et al., 2018). Stimulation of the lateral temporal cortex was done after feedback from neural recordings, which identified brain patterns regarding memory performance. The stimulation increased the probability of word recall and improved memory (Ezzyat et al., 2018). Thus, closed-loop stimulation allows the modulation and encoding of higher cognitive functions in real-time.

This brings us to the idea of “adaptive stimulation.” This is defined as a smart and adjustable stimulation method that is guided by signals from neural activity of specific cognitive functions, also called biomarkers (Kucewicz et al., 2022). Neural biomarkers are certain characteristics of neural activity that correlate with that specific cognitive process. For instance, in a large study that explores the relationship between electrical stimulation and its impact on memory performance and gamma activity during memory encoding, the gamma power is the biomarker for memory encoding. When stimulation increased gamma power in the lateral temporal cortex, it led to improved memory performance; on the other hand, when stimulation decreased gamma power in the mesial temporal lobe, it was associated with a decline in memory performance (Ezzyat et al., 2017; Goyal et al., 2018; Kucewicz et al., 2018). Identification of certain neural biomarkers that correlate to speech is essential for adaptive stimulation. Deep brain stimulation (DBS) therapy for Parkinson’s disease was among the initial utilization of this method. In this clinical study, oscillations within the beta frequency range act as biomarkers to regulate motor functions. In its foundational use of adaptive stimulation, these abnormal beta oscillations are identified in the captured signal, guiding the precise location and timing for therapeutic electrical stimulation (Little et al., 2013; Oyama et al., 2021). Adjustments can be made in real-time based on immediate localized assessment or retrospectively utilizing prolonged recordings transmitted from the embedded device. Although adaptive stimulation has been used for targeting pathological neural biomarkers correlated with motor dysfunction in Parkinson’s patients, this method can be adopted in encoding of new perceptions by analyzing biomarkers for higher cognitive functions and then targeting these physiological processes through stimulation. In combination with patient-specific brain modeling, which personalizes the encoding of new information in the brain by catering to the brain anatomy of each patient, closed-loop adaptive stimulation is a promising approach for encoding complex speech perceptions since it provides real-time decoding and feedback to the target electrode sites for optimal stimulation.

## 6 Discussion

Evoking speech-like perceptions through brain stimulation has promising prospects in the development of brain-computer interfaces and neural prostheses. Brain stimulation techniques could be used in conjunction with BCIs to enable direct communication between the brain and external speech devices, such as speech synthesizers or prosthetic vocal cords. By stimulating specific brain regions associated with speech production or perception, it may be possible to decode and synthesize speech signals for communication purposes. Optimizing brain stimulation techniques and controlling the perceptions that are induced may allow for communication without the need for external devices. This technology may provide a novel way of communication for patients with speech disorders such as aphasia or dysarthria. Although the prospects of brain stimulation in BCI are vast, there are many challenges to overcome before it can be used in clinical settings. Here, we propose some of the promising technologies that could be used for the development of speech BCI systems and speech encoding.

Multi-site and multi-parameter stimulation shows promise in evoking complex cognitive perceptions. Current stimulation methods cannot elicit speech-like perceptions or even simple auditory percepts in a consistent manner; traditional parameters that have been used for functional mapping and clinical research are not sufficient to induce desired speech-like percepts. A recent study reveals that the modulation of multi-site and multi-parameter stimulation techniques can be used to control and create diverse perceptions (Ryun et al., 2021; Hong et al., 2023). Changing the frequency during the stimulation trial, known as “Dynamic Frequency” stimulation, has shown promise in eliciting various somatosensory percepts during the stimulation of somatosensory regions (Ryun et al., 2021). Using this technique combined with various combinations of stimulation parameters (pulse width, current amplitude, frequency, and duration) and targeting multiple brain regions simultaneously may allow researchers to precisely control the quality of evoked perceptions. Recent investigation using MSS of the visual cortex has shown that stimulating at least two regions of the brain allows for the generation of a different number of phosphenes compared to single site (Bosking et al., 2022). Stimulation of three electrode sites evoked three distinct phosphenes more often (30 times) than stimulation of one (0 times) or two electrode sites (2 times). However, stimulation of four or more electrodes resulted in almost no phosphene generation. Although the encoding of higher cortical function still needs to be tested, this study shows that stimulation of up to three electrodes is most efficient when inducing simple visual percepts. Further investigation is needed to test the efficacy of stimulating more than two sites for the induction of perceptions that involve higher cortical functions, such as speech and memory.

The integration of multi-site stimulation (MSS) into personalized speech perception models offers significant advantages, particularly in inducing macro-level brain activation networks. Speech perception and other forms of perception involve extensive brain network activation, as evidenced by research showing that these processes engage broad neural networks. A direct cortical stimulation study of the somatosensory cortex illustrated how somatosensory perception activates widespread regions in the brain (Ryun et al., 2023). By manipulating and refining multi-electrode stimulation, it is proposed that the natural, distributed processing of speech can be replicated, thereby enhancing the overall effectiveness of the stimulation. Moreover, MSS may enhance the quality of perceptions by inducing independent percepts at multiple sites, especially in densely packed cortical areas. Although some studies in phosphene research have reported negative outcomes when stimulating more than three sites (Bosking et al., 2022), a recent MSS study for eliciting artificial somatosensation has shown to evoke multiple independent percepts (Ryun and Chung, 2024). Specifically, in this study, MSS not only elicits distinct perceptual responses but also modulates the quality of perception when applied to dense regions (Ryun and Chung, 2024). While further research is necessary to fully understand the underlying mechanisms of these effects, the initial results are promising. Therefore, MSS represents a robust approach to improving speech perception through personalized brain stimulation, combining extensive brain network engagement with enhanced perceptual quality.

**FIGURE 2:**
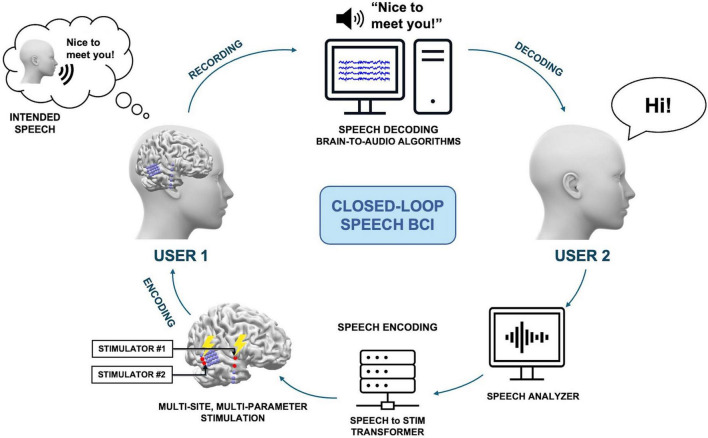
A proposed method of a closed-loop speech BCI system, allowing non-verbal communication between users. User 1 generates covert speech, which can be decoded with brain-to-audio speech decoders and synthesized into raw speech. Then, User 2 responds to the synthesized speech, and the subsequent speech is encoded into the brain of User 1.

Closed-loop BCIs, which use real-time feedback from brain activity to adjust stimulation parameters, have shown promise in enhancing the performance of invasive brain stimulation for inducing artificial speech-like perceptions. Closed-loop systems, in conjunction with adaptive stimulation paradigms, allow the optimization of stimulation strategies through precise, personalized approaches, potentially yielding superior therapeutic outcomes compared to open-loop stimulation. Figure 2 illustrates a proposed method of closed-loop speech BCI system designed to enhance speech perception through personalized multi-site and multi-parameter brain stimulation. The process begins with User 1 generating covert speech, which is decoded using brain-to-audio speech decoders and synthesized into raw speech, resulting in the phrase “Nice to meet you!” User 2 then responds to this synthesized speech by saying, “Hi!” The speech from User 2 is analyzed by an AI-based speech analyzer, which decodes the speech to extract relevant linguistic or sound features. These features are processed by a speech-to-stimulus transformer that utilizes a pre-established personalized speech perception brain model or dictionary. This model maps the extracted features to specific brain stimulation parameters. The system then employs multi-site, multi-parameter stimulation to induce the desired brain activity patterns for speech perception in User 1’s brain. The personalized brain model ensures that the stimulation parameters are tailored to the individual’s unique neural responses, enhancing the effectiveness of the stimulation. This closed-loop system iteratively refines the stimulation parameters based on real-time recording and feedback, ensuring optimal speech perception for both users. The integration of advanced AI analysis and personalized brain modeling makes this system a robust approach for improving speech perception through direct brain stimulation.

Given that the effects of stimulation can vary significantly based on brain states, specific brain regions, and pathological conditions, real-time monitoring of these neural signatures across different brain states in conjunction with stimulation can provide insights into ongoing network dynamics. Identifying these brain states and administering targeted stimulation at opportune moments and in the appropriate target areas holds great promise in the field of medicine and clinical research. This technology can increase efficacy, reduce side effects, and facilitate a deeper understanding of the underlying mechanisms. Currently, speech BCI research has been largely focused on speech production, specifically the production of acoustic sounds from neural recordings. However, language comprehension and production are both important to create a fully closed-loop BCI system, where neural data during speech is decoded and electrical stimulation is given to create speech-like perceptions. Further development of closed-loop systems can improve the accuracy and efficiency of speech perception in individuals with speech-related disorders. The combination of invasive brain stimulation with other techniques, such as brain imaging and machine learning, neural data analysis can enhance the efficacy and specificity of the induced speech-like percepts.

The practical implementation of brain models in experimental or clinical setups may involve several key stages. First, individual brain activity must be analyzed during speech perception and comprehension to extract unique features, representing a specific brain state. This involves using neuroimaging techniques such as fMRI, EEG, or magnetoencephalography (MEG) to capture and analyze these brain states. Second, brain stimulation parameters must be determined to induce the identified brain states. This process should consider both known physiological pathways and real-time monitoring of brain states to refine the stimulation parameters. Finally, the effectiveness of brain stimulation in achieving the desired perception should be validated experimentally. This involves iterating through steps of stimulation and monitoring, employing a trial-and-error approach to fine-tune the parameters. This process is applicable to both individuals with hearing impairments and those with normal hearing, with the approach tailored to the specific needs of the target population. For hearing-impaired individuals, the system compensates for their inability to hear sounds by directly stimulating the relevant brain regions involved in speech perception. For healthy individuals, the system can enhance or modify speech perception for therapeutic or research purposes. This iterative process of refining stimulation parameters based on feedback and observed outcomes is essential for building a robust, personalized brain stimulation model for speech perception. Through these steps, we aim to establish a reliable and effective personalized brain stimulation model for speech perception.

Overall, the future prospects for inducing artificial speech-like perception through invasive brain stimulation are promising, but further research is needed to optimize the technique for practical BCI systems. Current methods, such as multi-site, multi-parameter, and adaptive stimulation, present exciting avenues for the encoding of speech-like perception. To achieve a fully implantable closed-loop speech BCI system with bidirectional communication that includes both encoding and decoding, new strategies for stimulation need to be developed, and current techniques of MSS and closed-loop stimulation paradigms need to be optimized. For instance, newer parameters and stimulation technology need to be developed that mimics the natural neural activity during speech processing. Moreover, MSS needs to be investigated and explored to discover the optimal number and location of stimulation electrodes. Despite these challenges, we expect that MSS that takes into consideration the spatiotemporal patterns of neural activation, dynamic frequency and multi-parameter stimulation approaches may be promising tools for the input of artificial speech-like information in the human brain. Moreover, these emerging techniques of invasive stimulation may shed light on brain dynamics and lead to important insights into the mechanisms of speech processing and other higher cognitive functions. The input of speech-like percepts into the cerebral cortex can also be a crucial component of neuroprosthetic devices for restoring speech deficits. Encoding of speech information in the brain will not only benefit patients who lack the ability to communicate, but a fully implantable closed-loop speech BCI system will allow enhanced cognitive functions and restore other lost cortical functions as well.

## Author contribuitons

YH: Conceptualization, Investigation, Writing – original draft, Writing – review & editing. SR: Supervision, Validation, Writing – review & editing. CC: Conceptualization, Investigation, Supervision, Validation, Writing – original draft, Writing – review & editing.
